# A Mutual Capacitance Touch Readout IC with Synchronization in Touch and Mobile Display Driving for High Refresh Rate AMOLED Panels

**DOI:** 10.3390/mi12080922

**Published:** 2021-07-31

**Authors:** Seunghoon Ko

**Affiliations:** Department of Electronic Materials Engineering, Kwangwoon University, Seoul 01897, Korea; shko@kw.ac.kr

**Keywords:** touch screen panel, differential readout system, capacitance sensor

## Abstract

This paper presents a mutual capacitance touch readout IC architecture for 120 Hz high-refresh-rate AMOLED displays. In high-refresh-rate AMOLED panels, whole pixels in a horizontal line should be updated without any time-sharing with each other, leading to an amplified display noise on touch screen panel (TSP) electrodes. The proposed system architecture mitigates severe display noise by synchronizing the driving for the TSP and AMOLED pixel circuits. The proposed differential sensing technique, which is based on noise suppression in reference to mutual capacitance channels, minimizes common-mode display noise. In the front-end circuit, intrinsic circuit offset is cancelled by a chopping scheme, which correlates to the phase of the driving signals in the TSP driver and operating clocks of the front-end. Operating at a 120 Hz scan-rate, it reduces display noise by more than 11.6 dB when compared with the conventional single-ended TSP sensing method. With a built-in 130-nm CMOS, a prototype IC occupies an area of 8.02 mm^2^ while consuming 6.4-mW power from a 3.3 V analog voltage supply.

## 1. Introduction

Nowadays, with increasing demand for high-quality mobile device displays, display vendors strive to advance their technology featuring ultra-thin, transparent, and fast response time displays. Recent significant progress in mobile display driver integrated chips (MDDI) and pixel circuit technology results in the “seamless display”, or an introduction of a high refresh rate [1] in mobile devices where the MDDI updates each display frame within 8.33 milliseconds. In addition, a variable refresh rate (VRR) [2] is employed in high-end smartphones, in which the refresh rate is adjusted automatically depending on the display content. A refresh rate less than 10 Hz can be maintained for a static display image such as an always-on-display (AOD) function [3], while a fast frame transition of 120 Hz is needed for high-resolution gaming content.

**Figure 1:**
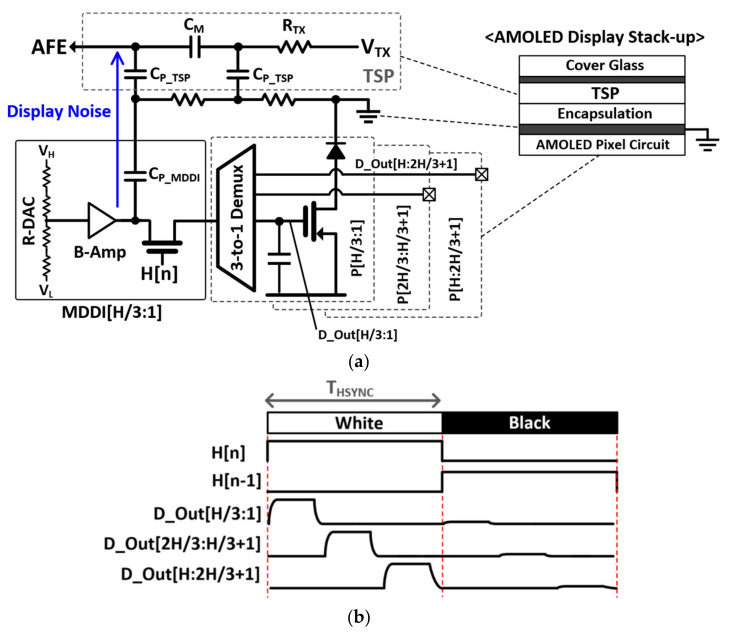
(**a**) An equivalent circuit consisting of an AMOLED, a TSP and an MDDI, and (**b**) a timing diagram of the MDDI operation using a 3-to-1 demultiplexer.

Figure 1a illustrates an equivalent circuit and the stack-up of an active-matrix organic light-emitting diode (AMOLED) pixel and touch screen panel (TSP) when MDDI operates. In an on-cell display panel [4], TSP electrodes are deposited and attached onto a display pixel layer using optically clear adhesive (OCA) in a separate manufacturing process. However, in case of AMOLED panels, TSP electrodes are directly printed over the encapsulation glass which protects AMOLED pixel from oxidation and physical damage. Therefore, the ground plane for AMOLED pixel should be shared with TSP readout system. Active shielding in the ground plane [4,5] cannot be applied to AMOLED display systems, which is more vulnerable to display noise and the effects of TSP parasitic capacitance.

The MDDI, consisting of a binary amplifier (B-Amp) and a resistive digital-to-analog converter (R-DAC), provides RGB signals, and the thin-film transistor circuit in the AMOLED receives and updates these signals into the array of pixels (P[H:1]). Demultiplexers (Demux) at the MDDI output have been widely adopted for low-resolution and low-refresh-rate displays. They ease the power and area budget of commercialized MDDIs. With a 3-to-1 Demux, the MDDI can refresh three times more pixels than each sub-block in the MDDI (MDDI[H/3:1]) during a horizontal blanking period (T_HSYNC_). Figure 1b illustrates the timing diagram of an MDDI when a zebra pattern repeating white and black lines is updated with a 3-to-1 Demux. During T_HSYNC_, the pixel update timing can be divided into 3 phases. For example, the demultiplexer outs of D_Out[H/3:1] are refreshed into the P[H/3:1] during one third of the T_HSYNC_. In contrast, for a 120 Hz refresh rate display such as full high-definition (FHD) or wide quad high-definition (WQHD) high-end displays having 1920 × 1080 and 2560 × 1440 resolution, there is no room for time-interleaving the AMOLED pixel driving during a T_HSYNC_. Column-parallel B-Amps and R-DACs per each AMOLED pixel should be integrated into the MDDI to update whole pixels (P[H:1]) in a horizontal line at the same time during a T_HSYNC_. Although this technique can enhance the refresh rate of a display update, substantial display interferences are also generated due to the simultaneous transition of outputs of MDDI[H:1]. These interferences are directly added to the TSP electrodes through the series of C_P_MDDI_ and C_P_TSP_, degrading the signal-to-noise ratio (SNR) when we measure the mutual capacitance (C_M_) between the transmitter (TX) and receiver (RX) TSP electrodes.

Therefore, high-resolution, high-refresh-rate displays lead to an increased burden for the analog front-end (AFE) of a TSP readout system and its post processing algorithm. Several researchers [6,7] have proposed using a frequency hopping scheme to avoid noisy frequency bands when choosing the TSP driving frequency. However, they would suffer from an in-band noise disturbance, the amplitude and frequency of which vary according to the display contents in a high-refresh-rate display. The works in [8,9,10,11] use differential front-end architecture to cancel out the common-mode interference, but the importance and difficulty of choosing a reference for capacitance reconstruction have not been addressed.

This article presents a column-parallel differential sensing TSP readout system with a noise-free capacitance reconstruction technique. It enables substantial noise reduction over the reference capacitance with synchronization of the display and TSP driving. The readout IC also introduces the use of different orthogonal sequences for differential and reference TSP capacitance sensing. The proposed readout system senses a total of 31 × 16 TSP mutual capacitances and operates at a 120 Hz TSP scan rate using a 3.3 V power supply. This article is organized as follows. Section 2 presents the operation principle of the proposed differential sensing architecture with the noise-free reference capacitance sensing technique. Section 3 presents the circuit implementation. The measurements, results, and discussion are provided in Section 4. Finally, Section 5 concludes this article.

## 2. Proposed Differential Sensing Scheme

### 2.1. Display Noise Measurement

**Figure 2:**
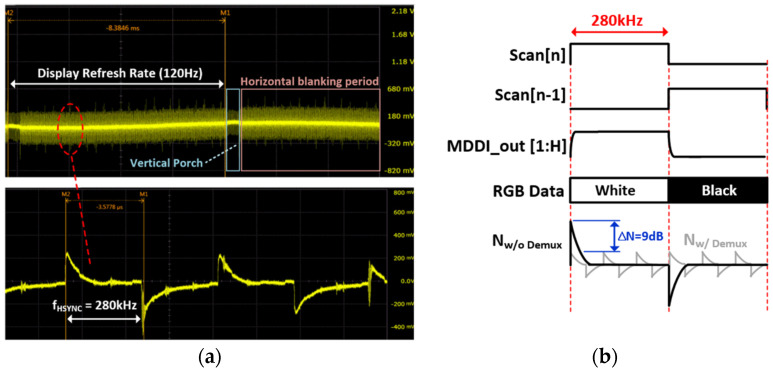
(**a**) Measured display noise with an FHD+ AMOLED display, and (**b**) comparison of display noise levels with a zebra image without Demux and with a 3-to-1 Demux configuration.

Figure 2 shows the display noise measured on a TSP electrode when a zebra pattern is updated onto a 2400 × 1080 FHD plus panel with a 120 Hz refresh rate. One horizontal (1-H) zebra pattern which alternates horizontal black and white colors at a time interval of T_HSYNC_ (=1/f_HSYNC_) was used for this measurement. In this case, we can approximately calculate the display noise frequency from the product of the refresh rate (120 Hz) and horizontal resolution (2400) of the panel. The maximum signal swing of the output of the MDDI (MDDI_ out[1:H]) occurs when it goes from black to white or from white to black horizontal lines. At those moments, display noise is also maximized. The noise waveform is a form of the derivative of a rectangular MDDI_Out, since the noise current flows across the series of C_P_MDDI_ and C_P_ TSP_ (Figure 1a). The harmonics of f_HSYNC_ also exist in the noise signal frequency. Note that the noise frequency highly depends on the display contents to be updated, so it cannot be expected or removed by the frequency hopping scheme proposed in prior works. Compared with the HD panel which executes time-interleaved updates using 3-to-1 Demux in a T_HSYNC_, the noise voltage increased by three times (9 dB), incurring a large noise penalty with a high-resolution, high-refresh-rate display.

### 2.2. Basic Principle of Differential Sensing

**Figure 3:**
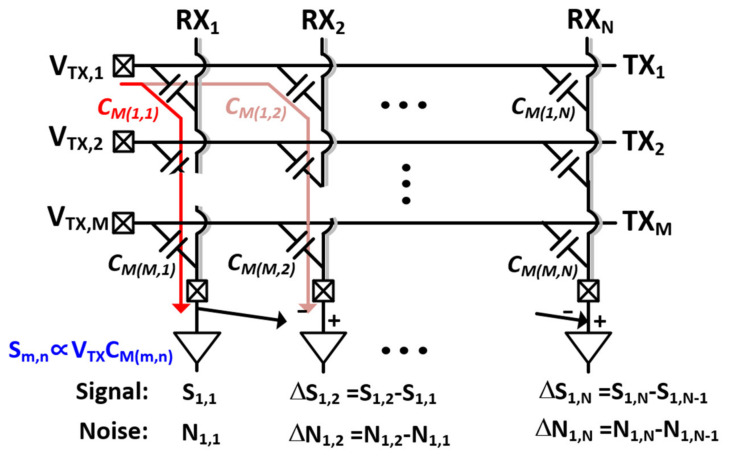
Illustration of the operation principle of a differential TSP sensing scheme.

The detailed operation of differential TSP sensing is shown in Figure 3. The TSP consists of M TX electrodes and N RX electrodes. An entire TSP channel is divided into the reference capacitance channels (C_M(1,1)_–C_M(M,1)_) in a, RX electrode, and the remaining capacitance channels (C_M(1,2)_–C_M(M,N)_) for differential sensing. The capacitance channel or mutual capacitance in a TSP is generated at the cross-section of each of the TX and RX electrodes. During the sensing phases for both differential and reference capacitance, the driver stimulates the TX electrodes (from TX_1_ to TX_M_) sequentially or concurrently using encoded TX signals (from V_TX,1_ to V_TX,M_). These signals are coupled through the mutual capacitance channel and measured at the RX electrode by the AFE. Therefore, touch sensitivity becomes proportional to the product of the mutual capacitance and the signal swing of the driver output. A simple dynamic buffer-generating square wave can be used as the TSP driver, reducing the design complexity. At the AFE, column-parallel differential sensing is usually employed to suppress common-mode noises, which include any external noises as well as the display interference. A fully or pseudo-differential trans-impedance amplifier or switched-capacitor integrator are used as a first AFE stage to detect only the capacitance difference (∆C_M_). At that moment, besides the ∆C_M,_ the interferences are frozen on the first stage but are cancelled differentially. Post-processing of capacitance data to extract an exact touch coordinate requires that the ∆C_M_ be recovered to a single-ended C_M_. We can execute an integral of the ∆C_M_ of a column in a horizonal direction, which is based on the reference capacitance channels C_M(1,1)_–C_M(M,1)_, in an RX electrode. Thus, the reference RX electrode acts like an integral basis and the recovered single-ended signals (*S_RCV_*(*m*, *n*)) for the C_M(m,n)_ can be expressed as

(1) $$S_{RCV}\left(m,n\right)=S_{m,1}+\gamma N_{m,1}+\overset{n}{\underset{k=2}{\sum }}\left(∆S_{m,k}+∆N_{m,k}\right)$$

where we assume that $S_{m,1}$ and $N_{m,1}$ are the capacitance and noise signals at the cross-section of the *m*th TX, and the 1st RX electrodes measured in a single-ended manner and $∆S_{m,k}$ and $∆N_{m,k}$ are the difference values of $S_{m,k}-S_{m,k-1}$ and $N_{m,k}-N_{m,k-1}$, respectively. The attenuation ratio (γ) of *N_m,_*_1_ needs special attention. If γ is equal to zero, $∆N_{m,k}$ can be suppressed by the differential operation. Otherwise, $N_{m,1}$ can be considered as a global noise component across all TSP channels in differential sensing.

This equation inspires us to choose and set the noise-immune reference capacitance channels, noise to which directly adds to the reconstructed capacitance value. Thus, the degree of noise suppression in a differential TSP sensing readout system depends on how much of the noise voltage is frozen in the reference capacitance channels.

### 2.3. Detailed Operation of the Proposed Scheme

**Figure 4:**
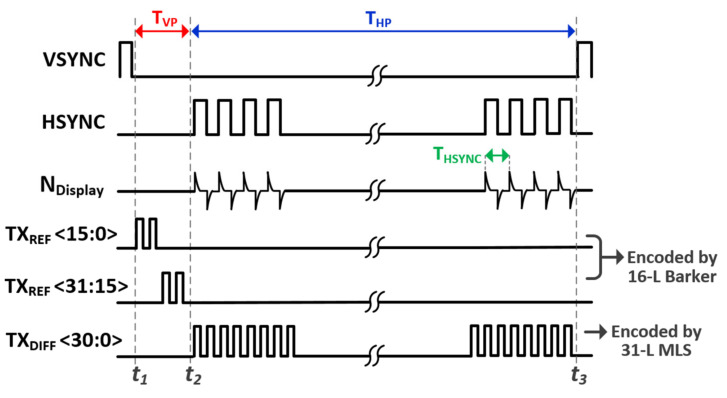
Timing diagram for illustrating the proposed differential TSP sensing scheme.

Figure 4 provides a timing diagram to illustrate our proposed differential TSP sensing mechanism. The display frame update repeats itself every vertical synchronization signal (VSYNC), where an entire MDDI operation cycle is divided into two phases: the vertical front porch (T_VP_) from t_1_ to t_2_, and the horizontal blanking period (T_HP_) from t_2_ to t_3_. During T_VP_, the voltage of the cathode plane in the AMOLED is changed, which, in a mobile device, is dependent on the brightness of the display. Since the rate of change of the voltage is too slow, the noise current from the cathode plane to the TSP electrode, which passes through the parasitic capacitance of TSP (C_P_TSP_) (Figure 1a), is also insignificant. The AMOLED pixel voltage begins to vary from t_2_. As with high-refresh-rate, high-resolution panels, all RGB pixels P[H:1] in a horizontal line should be updated in a T_HSYNC_, which is synchronous to the horizontal synchronization signal (HSYNC). In this case, the rising and falling time of the MDDI_out[H:1] should also decrease, leading to an amplified noise current insertion through the series of C_P_MDDI_ and C_P_ TSP_ to the TSP electrode. Total display noise is also proportional to the number of pixels (H) in a horizontal line to be updated. In this design, we take careful consideration in dealing with the timing of the reference and differential capacitance driving. The reference capacitance channels in an RX electrode are driven by TX_REF_ <30:0> during T_VP_. This is because the display noise is negligible compared with that during T_HP_. On the other hand, a column-parallel capacitance difference is measured during T_HP_. The common noise voltage freezes to both the positive and negative paths of the differential sensing AFE, but is cancelled differentially. By separating the sensing phases of the reference and differential capacitance, an improved SNR is achieved for its reference capacitance against the display noise, and sufficiently long T_HP_ helps to suppress any external noises [12,13] besides the display noise.

Different orthogonal sequences, TX_REF_ <30:0> and TX_DIFF_ <30:0>, were employed for the driving of the reference and differential capacitances. When we introduced the orthogonal driving into the TSP readout system [14,15,16], we faced a tradeoff between dynamic range and crosstalk. Although the multi-channel driven concept increased the sensing time for each TSP channel, leading to increased sensitivity, total capacitance to be processed by the AFE also increased. This limited the dynamic range of the AFE and increased the burden to the operation amplifier from the trans-impedance amplifier or the switched capacitor integrator, which are the first stage of AFE. For a Hadamard orthogonal sequence, the vertical code summation becomes equal to the number of orthogonal sequences. One method is to use another orthogonal sequence that minimizes the code summation, but it is at the expense of increased residue between the TSP channels in adjacent TX electrodes, where the residue can be defined by the inner product of orthogonal sequences. In case of the n-length (n-L) maximum-length orthogonal sequence (MLS) [17,18], the inner product is equal to n when inner product with itself is done. However, the inner product for different MLSs is reduced to 1, regardless of length. Thus, the crosstalk can be calculated to 1 divided by n, or by the code-length of the MLS.

**Figure 5:**
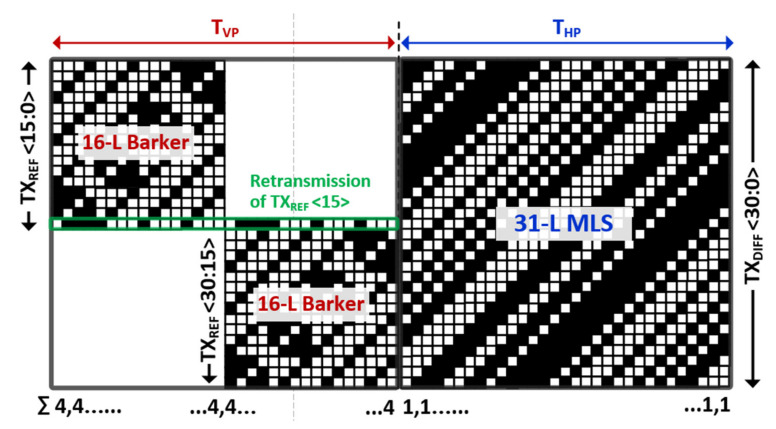
Application of different orthogonal sequences in reference and differential capacitance sensing.

In a practical design, when an FHD and an AMOLED having a 31 TX and 16 RX electrode TSP was evaluated, we applied a different orthogonal encoding method during the T_VP_ and T_HP_, as shown in Figure 5. Firstly, during T_VP_ for reference capacitance sensing, there are also two sensing phases: the upper TSP sensing phase, and the lower TSP sensing phase. The reasons are twofold. First, the panel bandwidth (f_TBW_UPPER_) of the upper TSP area is much smaller than that of the lower TSP area. Thus, the TX driver experiences the greatest delay when it transfers the charge of C_M(1,1)_ (in Figure 3) from TX [1] to RX [1]. This phase mismatch would disrupt the orthogonality if we were to choose a higher TX frequency free from external noises besides the display noise. Second, code summation should be minimized while maintaining perfect orthogonality between each TX electrode, which is highly desirable to set the integral basis for differential sensing. To achieve a reasonable tradeoff, a 16-length baker sequence [19,20] was applied to TX_REF_ <0:15> and TX_REF_ <15:31>. Here, TX_REF_ <15> was retransmitted for the lower TSP area, which can balance the code summation to four without incurring crosstalk.

During T_HP_, all TX electrodes are driven simultaneously by the 31-L MLSs, which is desirable for two reasons. First, display noise due to the RGB pixel updates in the T_HP_ does not saturate the AFE since the code summation is reduced to one mutual capacitance unit (C_M_). Second, the SNR increases by the square root of 31 compared with that of sequential driving. Although the TX frequency is restricted to be much lower than the f_TBW_UPPER_ and crosstalk exists between the TX electrodes, this noise penalty can be suppressed by permitting differential capacitance sensing.

In the TSP readout system, the inner product for TX_REF_ <30:0> and TX_DIFF_ <30:0> can be found to be

(2) $$TX_{\mathrm{REF}}<i>\;\cdot TX_{\mathrm{REF}}<j>=\left\{\begin{array}{c} 16\;\left(i=j\right)\\ 0\;\left(i\neq j\right) \end{array}\right.$$

(3) $$TX_{\mathrm{DIEF}}<i>\;\cdot TX_{\mathrm{DIEF}}<j>=\left\{\begin{array}{c} 31\;\left(i=j\right)\\ 1\;\left(i\neq j\right) \end{array}\right.$$

## 3. Circuit Implementations

### 3.1. Whole Readout System Architecture

**Figure 6:**
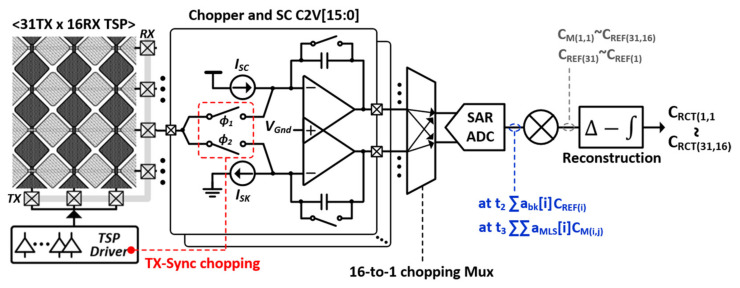
Proposed TSP-DDI synchronous differential front-end architecture.

Figure 6 shows the proposed TSP sensing architecture, where it senses the mutual capacitance of 31 TX electrodes and 16 RX-TSP electrodes. It is comprised of a TSP driver, a switched capacitor integrator for capacitance-to-voltage (C2V) conversion, a successive approximation register (SAR) analog-to-digital converter (ADC), and decoding and reconstruction logic. During the reference capacitance sensing phase (T_VP_), the TSP driver stimulates the TX electrode by TX_REF_ <30:0>. The TX carrier frequency is encoded by a 16-L Barker sequence, and the upper and lower TSP areas are driven separately. When the T_VP_ finishes, all TX electrodes are driven simultaneously by TX_DIFF_ <30:0>, which is encoded by 31-L MLS. To suppress external and display coupling noise, the sensing time should be sufficiently long, thus occupying most of the T_HP_ for differential sensing. At the same time, the front-end executes column-parallel TSP sensing with 16 C2V converters (C2V[15:0]). Besides conversion, a C2V converter acts as a discrete-time bandpass filter whose passband is centered on the TX frequency. The base capacitance, which is unchanged by the touch of a finger, is cancelled through a current compensation technique, where the sink (I_SK_) or source (I_SC_) current is provided to the AC-modulated capacitance signals at the C2V input. The voltage of the virtual ground of the C2V converter is fixed to half of the supply voltage, but exhibits an offset. In case of a panel having a large C_P_TSP_, it can saturate the output of a C2V. This risk can be removed by a chopping scheme where the phase of a TX signal is synchronous to the closing of ϕ_1_, ϕ_2_, and a 16-to-1 bidirectional ADC multiplexer (Mux).

The system adopts a differential sensing scheme processed in a digital domain. The reasons are twofold. First, in a practical design, we should sense both a single ended reference capacitance and the capacitance difference. Thus, we can choose the same input stage to sense both. Moreover, compared with previous work enabling higher-order band pass filtering with multiple AFE stages in an analog domain, it can ease the power and area budget of the IC with only two op-amps in the CDS. Second, compared with previous works, it can increase the sensing time by two times compared with differential AFE sensing. For simplicity of explanation, let us consider when the differences of C_M(1,1)_, C_M(1,2)_, and C_M(1,3)_ are sensed directly at the AFE. In this case, after the difference between C_M(1,1)_ and C_M(1,2)_ is sensed, that of C_M(1,2)_ and C_M(1,3)_ should be sensed sequentially to reconstruct the single-ended capacitance. This lowers the sensitivity by 3 dB compared with differential processing in the digital domain. This way, the insufficient noise filtering of differential sensing in digital domain can be supplemented. With a reference capacitance of C_REF(31)_–C_REF(1)_ and a single-ended measured capacitance of C_M(1,1)_–C_M(31,16)_, the final output corresponding to C_RCT(1,1)_–C_RCT(31,16)_ can be obtained.

### 3.2. Input Offset Cancellation and Capacitance Compensation Techniques

**Figure 7:**
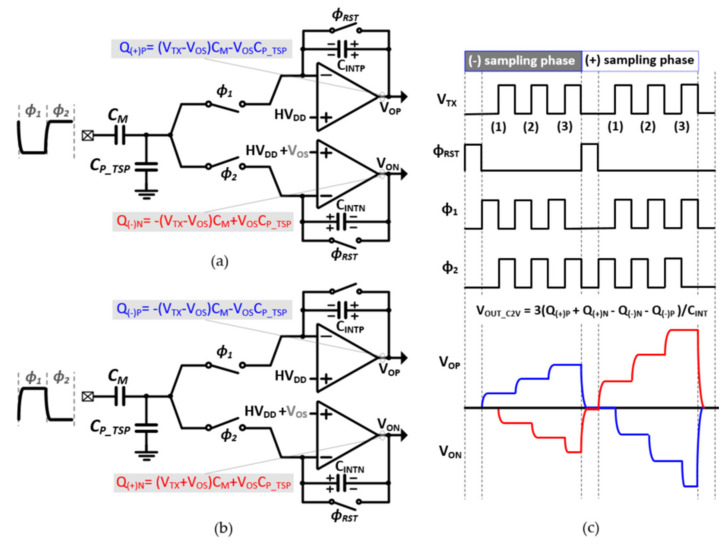
C2V configuration for (**a**) the negative offset sampling phase (ϕ_1_ = high, V_TX_ = low), (**b**) the positive offset sampling phase (ϕ_1_ = high, V_TX_ = high), and (**c**) its timing diagram.

Figure 7a–c shows the detailed operation of the proposed C2V converter with the input offset cancellation technique. The C2V converter consists of positive and negative switched capacitor (SC) integrators and sampling switches at the inputs. When ϕ_RST_ is high, the V_OP_ and V_ON_ are reset to half of supply voltage. As ϕ_RST_ falls, the charge on the mutual capacitance C_M_ is transferred to the integral capacitors of C_INTP_ and C_INTN_. When ϕ_1_ is high and V_TX_ is low, the charge proportional to C_M_V_TX_ is trapped on the ride-side of C_INTP_, increasing the V_OP_. Then, when both ϕ_2_ and V_TX_ are high, the negative charge of C_M_V_TX_ is transferred to the ride-side of C_INTN_, decreasing the V_ON_. By repeating itself every TX sample, moving-average bandpass filtering in the analog domain is obtained [21].

However, as the thickness of the AMOLED panel decreases, the TSP electrode becomes close to the display pixels, leading to the increased parasitic capacitance of C_P_TSP_. For encapsulation glass with a thickness of about 100 μm, the C_P_TSP_ is increased to tens of picofarads. In this case, C2V can saturate as the number of moving average taps increases. Let us consider that the (+) input of a positive SC integrator in the C2V converter is higher than that of the negative SC integrator by V_OS_. When ϕ_1_ is high, the C_P_TSP_ is charged to half of the supply voltage. Then, when ϕ_1_ is low and ϕ_2_ is high, the total charge on the C_P_TSP_ increases by C_P_TSP_V_OS_, bringing an equal positive charge to the right-hand side of C_INTN_. Since these offsets can be varied from C2V[15:0], it degrades sensing accuracy and saturates the AFE. For example, with 100 μF of C_P_TSP_, 2 μF of C_M_, 3, of TSP excitation signal, and 10, V of input offset voltage, an offset charge of 10 μC is also introduced, which is one-sixth of the charge from mutual capacitance. To minimize a circuit’s offset without any additional circuits, offset cancellation chopping is employed, which uses RX-correlated TSP driving. 

The sensing phase is divided into two phases: the positive (+) offset sampling phase and the negative (−) offset sampling phase. During the negative sampling phase, when ϕ_2_ is high, V_TX_ goes high. Therefore, a negative offset charge is stored on the C_INTP_ while positive (V_TX_–V_OS_)C_M_ is frozen on it, and vice versa for C_INTN_. The offset charge of V_OS_C_M_ is insignificant compared with that of V_OS_C_P_TSP_. During the positive sampling phase, when ϕ_1_ is high, V_TX_ goes high. In this case, the negatives of both the C_M_-charge and the offset charge are stored on the C_INTP_, and vice versa for C_INTN_ as well. To cancel the offset voltage at the C2V output, the V_OP_ in the positive sampling phase adds to the V_ON_ in the negative sampling phase. This can be enabled by reversing the connection in the 16-to-1 ADC Mux. The operational amplifier in the switched capacitor integrator is a two-stage class AB amplifier. Based on post-layout simulations, the amplifier achieves a DC gain of 61 dB and unity gain bandwidth of 12MHz while consuming 140 μW power from a 3.3 supply.

**Figure 8:**
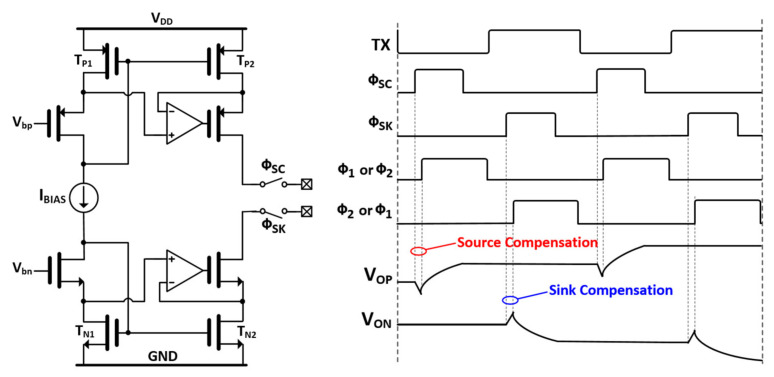
Circuit implementation of a capacitance compensator and its timing diagram.

During the sensing phase for both the T_VP_ and T_HP_, the base capacitance, which is not unchanged by a finger touch and occupies most of the dynamic range of AFE, should be removed. The detailed operation of the compensation circuit used here is shown in Figure 8 [20]. When ϕ_SC_ is high, the positive charge from the I_SC_ is provided to the negative input of a switched capacitor integrator. When ϕ_SK_ is high, the I_SK_, which is equal to the I_SC_, flows from its negative input. Since the TSP electrode is driven by AC signals, I_SK_ and I_SC_ repeat every high and low state of the TX signal. The DC bias current (I_BIAS_) is copied into the T_P1_ and T_N1_ and amplified N times by T_P2_ and T_N1_, respectively. In a practical design, a minimization of the mismatch between I_SK_ and I_SC_ is required. Thus, to minimize the channel length modulation effect, the V_DS_ of T_P1_ and T_P2_, and the V_DS_ of T_N1_ and T_N2_ are fixed to be equal using a regulated cascode feedback amplifier. Specifically, the phase of ϕ_SK_ or ϕ_SC_ leads that of ϕ_1_ or ϕ_2_, preventing a disturbance of the negative input voltage due to the charge transfer of C_M_ when compensation starts. In a practical design, the delay is fixed to a time duration of 5/f_MAIN_ where f_MAIN_ is 40 MHz or the operational clock of MCU in the designed IC.

## 4. Result and Discussion

**Figure 9:**
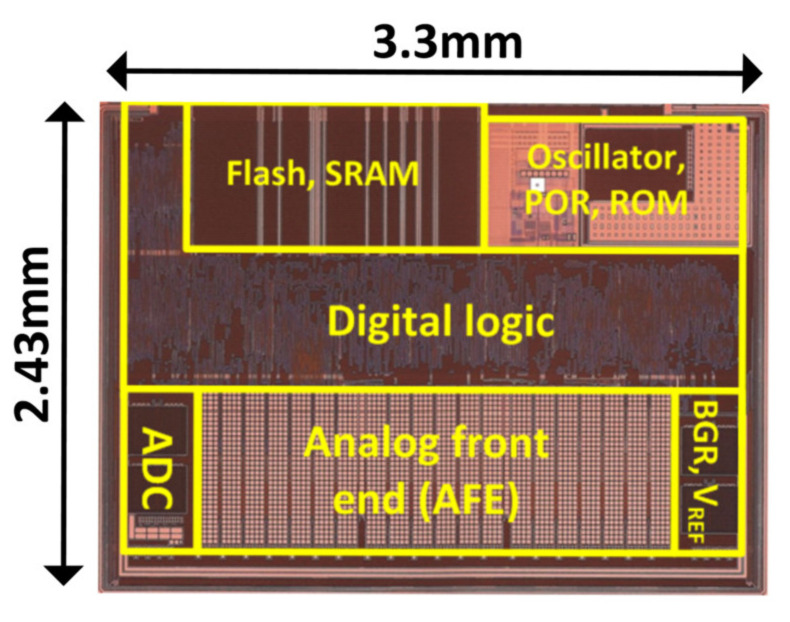
Die Photograph.

The proposed readout IC was fabricated in a 130-nm CMOS process. The die photograph is shown in Figure 9. The active area, which includes the AFE, SAR ADC, bandgap reference (BGR), and reference voltage generator (V_REF_), is 3.4 mm^2^ and the full chip area is 8.02 mm^2^. TSP driving was synchronized with the display update, where its frame rate is measured to be 123 Hz with a 2400 × 1080 FHD plus 6.1-inch AMOLED panel. For a TSP having 31 TX and 16 RX electrodes, the total power consumption was 17.1 W with a 3.3, power supply. The power breakdown for the AFE was 6.4 W. Operating the AFE only during part of a frame reduced its power consumption. Here, the AFE scan time was reduced to 3 ms, including the reference capacitance and differential capacitance sensing phases.

**Figure 10:**
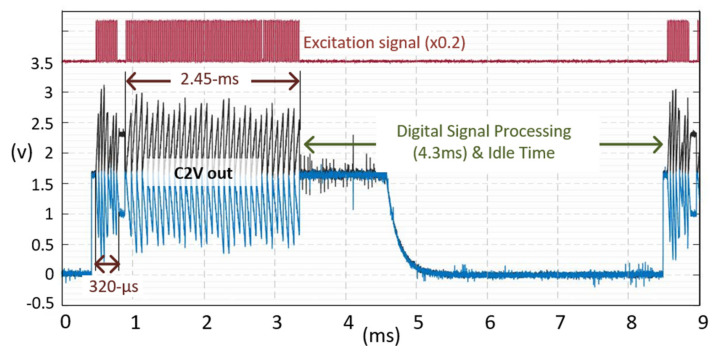
Measured waveforms of the TX signal (red) and the C2V output (black and blue) when synchronizing TSP and MDDI driving.

**Figure 11:**
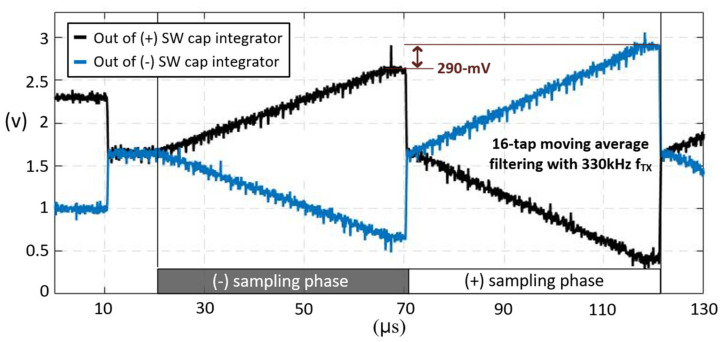
Measured waveforms of the C2V output when the chopping scheme is applied.

**Figure 12:**
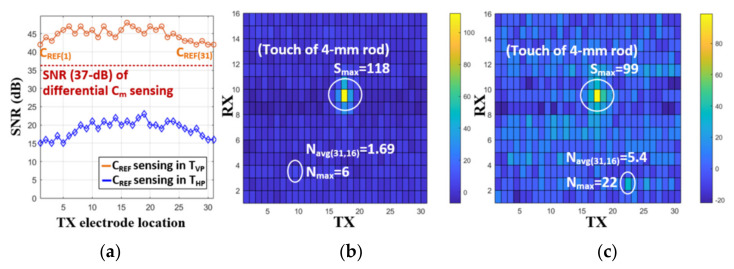
(**a**) Comparison of SNR for reference capacitance (C_REF_) during T_VP_ and T_HP_, and the measured RMS touch profile of (**b**) the proposed differential TSP sensing and (**c**) conventional single-ended sensing.

Figure 10 shows the measured TX signal and C2V output in a TSP frame. The sensing phase for reference capacitance sensing was 320 μs, while the differential sensing time was set to be 2.45 ms. Since touch coordinates should be extracted and provided to the application processor in a smartphone every TSP frame (123 Hz), digital signal processing time for interpolation, coordinate filtering, and baseline calibration is needed, and was set to be 4.3 ms. Figure 11 provides the measured C2V output before the 16-to-1 ADC multiplexer when the input offset cancellation technique was applied. The TX frequency of 330 kHz and a 16-repetition of the TX pulse were used for measurement. One orthogonal instant was divided into (+) and (−) sampling phases. The output voltage difference or offset voltage between these phases was 290 mV, which would be eliminated by adding outputs in each phase. To evaluate the effectiveness of sensing in T_VP_, the reference capacitance (C_ref(1)_~C_ref(31)_) was measured during both T_VP_ and T_HP_, as shown in Figure 12a where the 1-H zebra image was updated. The SNR measured in the T_VP_ was 22 dB higher than that of the T_HP_, since any display signals related to the 1-H zebra image did not exist during T_VP_. Figure 12b,c shows the differential and single-ended measured touch profile when a 4 mm diameter conductive rod was touched, with a display update from 1-H zebra image. Here, the touch data was RMS-averaged over 1000-frames. The noise values of the T_HP_ (N_max_, N_avg(31,16)_) were more than three times larger than that of the T_VP_. The SNR for differential sensing was measured to 37 dB, while that of single-ended sensing was 25.4 dB. The SNR loss between the reference capacitance and the differential capacitance sensing was 9 dB (Figure 12a). This is because the coupled display noises at each RX electrode have a mismatch in phase and amplitude. The performance summary and comparison with previous works are shown in Table 1. Unlike other state-of-the-art works, it describes noise-immunity to display noise and suppresses it by 37 dB in the worst possible case. Although other works achieved better SNR, a metal-mesh TSP sensor has higher capacitive change according to touch. The metal-mesh sensor also has smaller TSP-series resistance, allowing the use of a higher TSP frequency.

## 5. Conclusions

This article describes a display-noise-immune TSP readout system with synchronization of touch and display driving. It reduces the display noise by more than 4 times and has a compact structure without any additional circuits for differential sensing. The tradeoff between dynamic range and crosstalk is eased by introducing different orthogonal encoding for reference and differential sensing.

This technique is applicable to other display architectures. It can be easily adopted in an LCD or flexible AMOLED where the vertical porch and horizontal update instants exist.

## Figures and Tables

**Table 1:** Performance summary and comparison to previous works.

	This Work	Ref. [14]	Ref. [15]	Ref. [22]
Process	130 nm	180 nm	180 nm	350 nm
Electrodes	31 TX and 16 RX	16 TX and 33 RX	36 TX and 64 RX	15 TX and 10 RX
Scan rate	330 Hz (Synchronous to 120 Hz AMOLED)	120Hz	85~385Hz	250 Hz
Power Consumption	6.4 mW (AFE) 17.1 mW (Full chip)	17.8 mW	67.7 mW	12.8 mW
Chip area	3.4 mm^2^ (AFE) 8.02 mm^2^ (Full chip)	7.1 mm^2^	36 mm^2^	4.89 mm^2^
SNR	46 dB without display noise 37 dB with zebra pattern in 120 Hz AMOLED	57 dB	54 dB	43 dB
TSP Type	ITO	Metal Mesh	ITO	Metal Mesh
